# Log-Gabor Energy Based Multimodal Medical Image Fusion in NSCT Domain

**DOI:** 10.1155/2014/835481

**Published:** 2014-08-24

**Authors:** Yong Yang, Song Tong, Shuying Huang, Pan Lin

**Affiliations:** ^1^School of Information Technology, Jiangxi University of Finance and Economics, Nanchang 330032, China; ^2^School of Software and Communication Engineering, Jiangxi University of Finance and Economics, Nanchang 330032, China; ^3^Institute of Biomedical Engineering, Xi'an Jiaotong University, Xi'an 710049, China

## Abstract

Multimodal medical image fusion is a powerful tool in clinical applications such as noninvasive diagnosis, image-guided radiotherapy, and treatment planning. In this paper, a novel nonsubsampled Contourlet transform (NSCT) based method for multimodal medical image fusion is presented, which is approximately shift invariant and can effectively suppress the pseudo-Gibbs phenomena. The source medical images are initially transformed by NSCT followed by fusing low- and high-frequency components. The phase congruency that can provide a contrast and brightness-invariant representation is applied to fuse low-frequency coefficients, whereas the Log-Gabor energy that can efficiently determine the frequency coefficients from the clear and detail parts is employed to fuse the high-frequency coefficients. The proposed fusion method has been compared with the discrete wavelet transform (DWT), the fast discrete curvelet transform (FDCT), and the dual tree complex wavelet transform (DTCWT) based image fusion methods and other NSCT-based methods. Visually and quantitatively experimental results indicate that the proposed fusion method can obtain more effective and accurate fusion results of multimodal medical images than other algorithms. Further, the applicability of the proposed method has been testified by carrying out a clinical example on a woman affected with recurrent tumor images.

## 1. Introduction

Medical imaging has attracted increasing attention in the recent years due to its vital component in medical diagnostics and treatment [1]. However, each imaging modality reports on a restricted domain and provides information in limited domains that some are common, and some are unique [2]. For example, computed tomography (CT) image can provide dense structures like bones and hard tissues with less distortion whereas magnetic resonance imaging (MRI) image is better visualized in the case of soft tissues [3]. Similarly, T1-MRI image provides the details of an anatomical structure of tissues while T2-MRI image provides information about normal and pathological tissues [4]. As a result, multimodal medical images which have relevant and complementary information are necessary to be combined for a compendious figure [5]. The multimodal medical image fusion is the possible way to integrate complementary information from multiple modality images [6]. The image fusion not only obtains a more accurate and complete description of the same target, but also reduces the randomness and redundancy to increase the clinical applicability of image-guided diagnosis and assessment of medical problems [7].

Generally image fusion techniques can be divided into spatial domain and frequency domain techniques [8]. Spatial domain techniques are carried out directly on the source images. Weighted average method is the simplest spatial domain approach. However, along with simplicity, this method leads to several undesirable side effects like reduced contrast [9]. Other spatial domain techniques have been developed, such as intensity-hue-saturation (IHS), principal component analysis (PCA), and the Brovey transform [10]. Although the fused images obtained by these methods have high spatial quality, they usually overlook the high quality of spectral information and suffer from spectral degradation [10]. Li et al. [11] introduced the artificial neural network (ANN) to make image fusion. However, the performance of ANN depends on the sample images and this is not an appealing characteristic. Yang and Blum [12] used a statistical approach to fuse the images. In their method, the distortion is modeled as a mixture of Gaussian probability density functions which is a limiting assumption. Since the actual objects usually contain structures at many different scales or resolutions and multiscale techniques can provide the means to exploit this fact, the frequency domain techniques especially the multiscale techniques have attracted more and more interest in image fusion [13].

In frequency domain techniques, each source image is first decomposed into a sequence of multiscale coefficients. Various fusion rules are then employed in the selection of these coefficients, which are synthesized via inverse transforms to form the fused image. Recently, series of frequency domain methods have been explored by using multiscale transform, including Laplacian pyramid transform, gradient pyramid transform, filter-subtract-decimate pyramid transform, discrete wavelet transform (DWT), and complex wavelet transform (CWT) [14–20]. There is evidence that multiscale transform based signal decomposition is similar to the human visual system. As we know the wavelet analysis, with its upstanding localize peculiarity in both time and frequency domain, has become one of the most commonly used methods in the fields of multiscale transform based image fusion [16]. However, wavelet analysis cannot effectively represent the line singularities and plane singularities of the images and thus cannot represent the directions of the edges of images accurately. To overcome these shortcomings of the wavelet transform, Do and Vetterli [17] proposed Contourlet transform which can give the asymptotic optimal representation of contours and has been successfully used for image fusion. However, the up- and downsampling process of Contourlet decomposition and reconstruction results in the Contourlet transform lacking shift-invariance and having pseudo-Gibbs phenomena in the fused image [19]. Later, da Cunha et al. [20] proposed the nonsubsampled Contourlet transform (NSCT) based on Contourlet transform. This method inherits the advantages of Contourlet transform, while possessing shift-invariance and effectively suppressing pseudo-Gibbs phenomena.

Although quite good results have been reported by NSCT based method, there is still much room to improve the fusion performance in the coefficient selection as follows.

- The low-frequency coefficients of the fused image can be simply acquired by averaging the low-frequency coefficients of the input images. This rule decreased contrast in the fused images [21] and cannot give the fused subimage of high quality for medical images.
- The popularly used larger absolute rule is implemented in the value of a single pixel of the current high-frequency subband. The disadvantage of this method is that the coefficients only know the value of a single pixel but not any of the relationship between the corresponding coefficients in high-frequency subbands [22].
- Most fusion rules of the NSCT-based methods are implemented in multifocus images [23], remote sensing images [24], and infrared and visible images [25]. The results are not of the same quality as those of the multimodal medical images. For example, Chai et al. [22] proposed a NSCT method based on features contrast of multiscale products to fuse multifocus images. However, it has been proven that this algorithm is not able to utilize prominent information present in the subbands efficiently and results in the poor quality when it is used to fuse multimodal medical images [26].

In this paper, a novel fusion framework based on NSCT is proposed for multimodal medical images. The main contribution of the method lies in the proposed fusion rule, which can capture the best membership of source images' coefficients to the corresponding fused coefficient. The phase congruency and Log-Gabor energy are unified as the fusion rules for low- and high-frequency coefficients, respectively. The phase congruency provides a contrast and brightness-invariant representation of low-frequency coefficients whereas Log-Gabor energy efficiently determines the frequency coefficients from the clear and detail parts in the high frequency. The combinations of these two techniques can preserve more details from source images and thus improve the quality of the fused images. Experiments indicate that the proposed framework can provide a better fusion outcome when compared to series of traditional image fusion methods in terms of both subjective and objective evaluations.

The rest of the paper is organized as follows. NSCT and phase congruency are described in Section 2 followed by the proposed multimodal medical image fusion framework in Section 3. Experimental results and discussions are given in Section 4 and the concluding remarks are presented in Section 5.

## 2. Preliminaries

This section provides the description of concepts on which the proposed framework is based. These concepts, including NSCT and phase congruency, are described as follows.

### 2.1. Nonsubsampled Contourlet Transform (NSCT)

**Figure 1:**
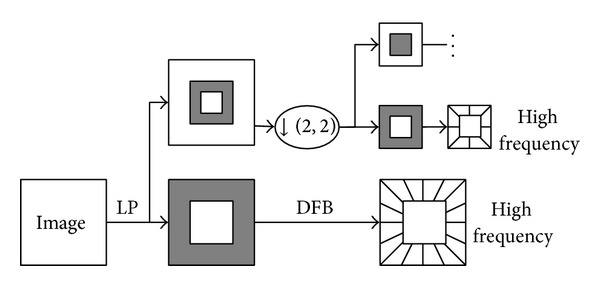
Contourlet decomposed schematic diagram.

Contourlet transform can be divided into two stages [19]: Laplacian pyramid (LP) and directional filter bank (DFB) and offers an efficient directional multiresolution image representation. Among them, LP is first utilized to capture the point singularities and then followed by the DFB to link the singular point into linear structures. LP is used to decompose the original images into low-frequency and high-frequency subimages, and DFB divides the high-frequency subbands into directional subbands. The Contourlet decomposed schematic diagram is shown in Figure 1.

**Figure 2:**
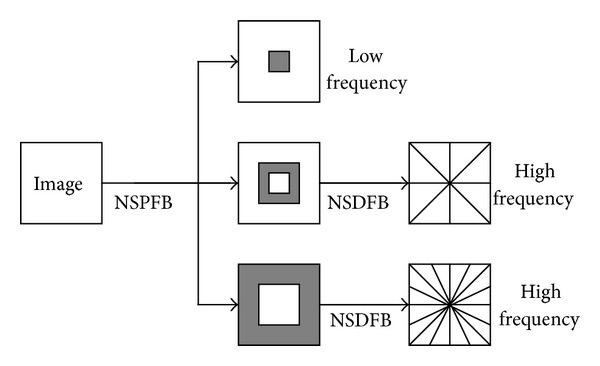
NSCT decomposed schematic diagram.

The NSCT is proposed based on the theory of Contourlet transform. NSCT inherits the advantage of Contourlet transform, enhances directional selectivity and shift-invariance, and effectively overcomes the pseudo-Gibbs phenomena. NSCT is built on nonsubsampled pyramid filter bank (NSPFB) and nonsubsampled directional filter bank (NSDFB) [21].  Figure 2 gives the NSCT decomposition framework with *k* = 2 levels.

The NSPFB ensures the multiscale performance by taking advantage of a two-channel nonsubsampled filter bank and one low-frequency subband and one high-frequency subband that can be produced at each decomposition level. The NSDFB is a two-channel nonsubsampled filter bank constructed by eliminating the downsamplers and upsamplers and combining the directional fan filter banks in the nonsubsampled directional filter [23]. NSDFB allows the direction decomposition with *l* levels in each high-frequency subbands from NSPFB and then produces 2^*l*^ directional subbands with the same size as the source images. Thus, the NSDFB provides the NSCT the multidirection performance and offers more precise directional detail information to get more accurate results [23]. Therefore, NSCT leads to better frequency selectivity and an essential property of the shift-invariance on account of nonsubsampled operation. The size of different subimages decomposed by NSCT is identical. Additionally, NSCT-based image fusion can effectively mitigate the effects of misregistration on the results [27]. Therefore, NSCT is more suitable for image fusion.

### 2.2. Phase Congruency

Phase congruency is a feature perception approach which provides information that is invariant to image illumination and contrast [28]. This model is built on the Local Energy Model [29], which postulates that important features can be found at points where the Fourier components are maximally in phase. Furthermore, the angle at which the phase congruency occurs signifies the feature type. The phase congruency can be used for feature detection [30]. The model provides useful feature localization and noise compensation. The phase congruency at a point (*i*, *j*) can be defined as follows [31]:

(1) PCθ(i,j) =∑nWθ(i,j)[Anθ(i,j)(cos⁡(ϕnθ(i,j)−ϕ−nθ(i,j)))         −|sin(ϕnθ(i,j)−ϕ−nθ(i,j))|−T]+  ×(∑nAnθ(i,j)+ε)−1,

where *θ* is the orientation, *W* ^*θ*^(*i*, *j*) is the weighting factor based on frequency spread, *A*_*n*_^*θ*^(*i*, *j*) and ${\overset{-}{\phi }}_{n}^{\theta }(i,j)$ are the amplitude and phase for wavelet scale *n*, respectively, *ϕ*_*n*_^*θ*^(*i*, *j*) is the weighted mean phase, *T* is a noise threshold constant, and *ε* is a small constant value to avoid division by zero. The notation []_+_ denotes that the enclosed quantity is equal to itself when the value is positive, and zero otherwise. For details of phase congruency measure see [29].

As we know the multimodal medical images have the following characteristics:

- the images of different modal have significantly different pixel mappings;
- the capturing environment of different modalities varies and resulted in the change of illumination and contrast;
- the edges and corners in the images are identified by collecting frequency components of the image that is in phase.

According to the literature [26, 32], it is easy to find that phase congruency is not only invariant to different pixel intensity mappings, illumination, and contrast changes, but also gives the Fourier components that are maximally in phase. These all will lead to efficient fusion. That is why we use phase congruency for multimodal medical fusion.

## 3. Proposed Multimodal Medical Image Fusion Framework

**Figure 3:**
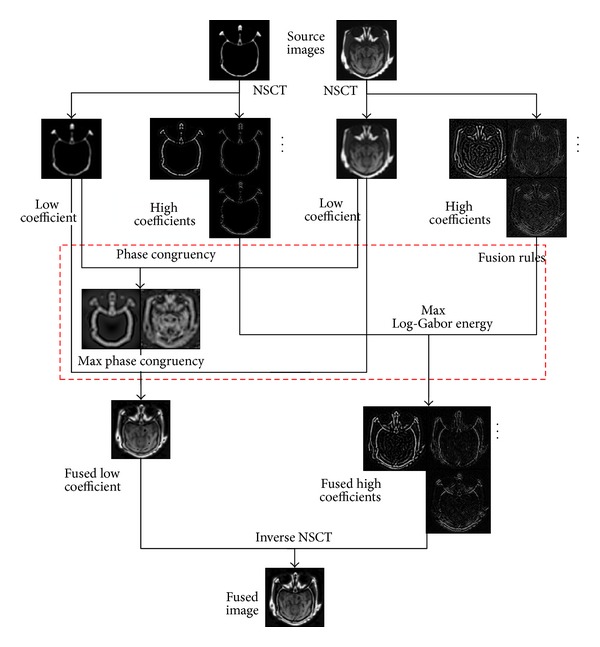
The framework of the proposed fusion algorithm.

The framework of the proposed multimodal medical image fusion algorithm is depicted in Figure 3, but before describing it, the definition of local Log-Gabor energy in NSCT domain is first described as follows.

### 3.1. Log-Gabor Energy in NSCT Domain

The high-frequency coefficients in NSCT domain represent the detailed components of the source images, such as the edges, textures, and region boundaries [21]. In general, the coefficients with larger absolute values correspond to the sharper brightness in the image. It is to be noted that the noise is also related to high-frequency coefficients and may cause miscalculation of sharpness values and, therefore, affect the fusion performance [26]. Furthermore, human visual system is generally more sensitive to texture detail features than the value of a single pixel.

To overcome the defect mentioned above, a novel high-frequency fusion rule based on local Log-Gabor energy is designed in this paper. Gabor wavelet is a popular technique that has been extensively used to extract texture features [33]. Log-Gabor filters are proposed based on Gabor filters. Compared with Gabor filters, Log-Gabor filters cover the shortage of the high frequency of Gabor filter component expression and more in accord with human visual system [34]. Therefore, Log-Gabor wavelet can achieve optimal spatial orientation and wider spectrum information at the same time and thus more truly reflect the frequency response of the natural images and improve the performance in terms of the accuracy [35].

Under polar coordinates, the Log-Gabor wavelet is expressed as follows [36]:

(2) $$\begin{array}{c} g\left(f,\theta \right)=\exp\left\{-\frac{{\left[\ln\left(f/f_{0}\right)\right]}^{2}}{2{\left[\ln\left(\sigma /f_{0}\right)\right]}^{2}}\right\}\times \exp\left\{-\frac{{\left(\theta -\theta_{0}\right)}^{2}}{2\sigma_{\theta }^{2}}\right\}, \end{array}$$

in which *f*_0_ is the center frequency of the Log-Gabor filter, *θ*_0_ is the direction of the filter, *σ* is used to determine the bandwidth of the radial filter, and *σ*_*θ*_ is used to determine the bandwidth of the orientation. If *g*_*kl*_^*uv*^(*i*, *j*) correspond to Log-Gabor wavelets in scale *u* and direction *v*, the signal response is expressed as follows:

(3) $$\begin{array}{c} G_{kl}^{uv}\left(i,j\right)=H_{kl}\left(i,j\right)*g_{kl}^{uv}\left(i,j\right), \end{array}$$

where *H*_*kl*_(*i*, *j*) is the coefficient located at (*i*, *j*) in high-frequency subimages of the source image *A* or *B* at the *k*th scale, *l*th direction, and ∗ denotes convolution operation. The Log-Gabor energy of high-frequency subimages at the *k*th scale, *l*th direction, is expressed as follows:

(4) $$\begin{array}{c} E_{kl}\left(i,j\right)=\overset{U}{\underset{u=1}{\sum }}\overset{V}{\underset{v=1}{\sum }}\sqrt{\text{real}{\left(G_{kl}^{uv}\left(i,j\right)\right)}^{2}+\text{imag}{\left(G_{kl}^{uv}\left(i,j\right)\right)}^{2}}, \end{array}$$

in which, real(*G*_*kl*_^*uv*^(*i*, *j*)) is the real part of *G*_*kl*_^*uv*^(*i*, *j*) and imag(*G*_*kl*_^*uv*^(*i*, *j*)) is the imaginary part of *G*_*kl*_^*uv*^(*i*, *j*). The Log-Gabor energy in NSCT domain at the local area around the pixel (*i*, *j*) is given as

(5) $$\begin{array}{c} \text{L}{\text{E}}_{kl}\left(i,j\right)=\frac{1}{\left(2M+1\right)\left(2N+1\right)}\overset{M}{\underset{m=-M\;}{\sum }}\overset{N}{\underset{n=-N}{\sum }}E_{kl}\left(i+m,j+n\right), \end{array}$$

in which (2*M* + 1)(2*N* + 1) is the window size. The proposed definition of the local Log-Gabor energy not only extracts more useful features from high-frequency coefficients, but also keeps a well performance in noisy environment.

### 3.2. Proposed Fusion Framework

The proposed NSCT-based image fusion framework is discussed in this subsection. Considering the input multimodal medical images (*A* and *B*) are perfectly registered. The framework of the proposed fusion method is shown Figure 3 and described as the following three steps.

**Step 1 .**

Perform *θ*-level NSCT on *A* and *B* to obtain one low-frequency subimage and series of high-frequency subimages at each level and direction *l*; that is, *A* : {*L* ^*A*^, *H*_*k*,*l*_^*A*^} and *B* : {*L* ^*B*^, *H*_*k*,*l*_^*B*^}, where *L* ^*A*^, *L* ^*B*^ are the low-frequency subimages and *H*_*k*,*l*_^*A*^, *H*_*k*,*l*_^*A*^ represent the high-frequency subimages at level *k* ∈ [1, *θ*] in the orientation *l*.

**Step 2 .**

Fuse low- and high-frequency subbands via the following novel fusion rule to obtain composite low- and high-frequency subbands.

The low-frequency coefficients represent the approximation component of the source images. The popular widely used approach is to apply the averaging methods to produce the fused coefficients. However, this rule reduced contrast in the fused images and cannot give the fused subimage of high quality for medical image. Therefore, the criterion based on the phase congruency that is introduced in Section 2.2 is employed to fuse the low-frequency coefficients. The fusion rule for the low-frequency subbands is defined as

(6) LF(i,j)={LA(i,j),if  PCAθ(i,j)>PCBθ(i,j)LB(i,j),if  PCAθ(i,j)<PCBθ(i,j)0.5(LA(i,j)+LB(i,j)),if  PCAθ(i,j)=PCBθ(i,j),

where PC^*Aθ*^(*i*, *j*), PC^*Bθ*^(*i*, *j*) is the phase congruency extracted from low-frequency subimages of the source images *A* and *B*, respectively.

For the high-frequency coefficients, the most common fusion rule is selecting the coefficient with larger absolute values. This rule does not take any consideration of the surrounding pixels and cannot give the fused components of high quality for medical image. Especially when the source images contain noise, the noise could be mistaken for fused coefficients and cause miscalculation of the sharpness value. Therefore, the criterion based on Log-Gabor energy is introduced to fuse high-frequency coefficients. The fusion rule for the high-frequency subbands is defined as

(7) $$\begin{array}{c} H_{kl}^{F}\left(i,j\right)=\{\begin{array}{cc} H_{kl}^{A}\left(i,j\right), & \begin{array}{c} \text{if}\;\;\text{L}{\text{E}}_{kl}^{A}\left(i,j\right)\geq \text{L}{\text{E}}_{kl}^{B}\left(i,j\right) \end{array}\\ H_{kl}^{B}\left(i,j\right), & \text{otherwise}, \end{array} \end{array}$$

where LE_*kl*_^*A*^(*i*, *j*) and LE_*kl*_^*B*^(*i*, *j*) are the local Log-Gabor energy extracted from high-frequency subimages at the *k*th scale and *l*th direction of source images *A* and *B*, respectively.

**Step 3 .**

Perform *θ*-level by the inverse NSCT on the fused low- and high-frequency subimages. The fused image is obtained ultimately in this way.

## 4. The Experimental Results and Analysis

It is well known that different image quality metrics imply the visual quality of images from different aspects, but none of them can directly imply the quality. In this paper, we consider both the visual representation and quantitative assessment of the fused images. For evaluation of the proposed fusion method, we have considered five separate fusion performance metrics defined as below.

### 4.1. Evaluation Index System

#### 4.1.1. Standard Deviation

The standard deviation (STD) of an image with size of *M*_0_ × *N*_0_ is defined as [37]:

(8) $$\begin{array}{c} \text{Std}={\left(\frac{1}{M_{0}\times N_{0}}\overset{M_{0}}{\underset{i=1}{\sum }}\overset{N_{0}}{\underset{j=1}{\sum }}{\left(F\left(i,j\right)-\hat{\mu }\right)}^{2}\right)}^{1/2}, \end{array}$$

where *F*(*i*, *j*) is the pixel value of the fused image at the position (*i*, *j*) and $\hat{\mu }$ is the mean value of the image. The STD can be used to estimate how widely the gray values spread in an image. The larger the STD, the better the result.

#### 4.1.2. Edge Based Similarity Measure

The edge based similarity measure (*Q* ^*AB*/*F*^) is proposed by Xydeas and Petrović [38]. The definition is given as

(9) QAB/F =∑i=1M0∑j=1N0[QAF(i,j)wA(i,j)+QBF(i,j)wB(i,j)]∑i=1M0∑j=1N0[wA(i,j)+wB(i,j)],

where *w* ^*A*^(*i*, *j*) and *w* ^*B*^(*i*, *j*) are the corresponding gradient strength for images *A* and *B*, respectively. The definition of *Q* ^*AF*^(*i*, *j*) and *Q* ^*BF*^(*i*, *j*) is given as

(10) $$\begin{array}{c} Q^{AF}\left(i,j\right)=Q_{a}^{AF}\left(i,j\right)Q_{g}^{AF}\left(i,j\right),\\ Q^{BF}\left(i,j\right)=Q_{a}^{BF}\left(i,j\right)Q_{g}^{BF}\left(i,j\right), \end{array}$$

where *Q*_*a*_^*xF*^(*i*, *j*) and *Q*_*g*_^*xF*^(*i*, *j*) are the edge strength and orientation preservation values at location for image *x* (*A* or *B*), respectively. The edge based similarity measure gives the similarity between the edges transferred from the input images to the fused image [26]. The larger the value, the better the fusion result.

#### 4.1.3. Mutual Information

Mutual information (MI) [39] between the fusion image *F* and the source images *A* and *B* is defined as follows:

(11) $$\begin{array}{c} \text{MI}=\text{M}{\text{I}}^{AF}+\text{M}{\text{I}}^{BF},\\ \text{M}{\text{I}}^{AF}=\overset{L}{\underset{f=0}{\sum }}\overset{L}{\underset{a=0}{\sum }}p^{AF}\left(a,f\right)\text{lo}{\text{g}}_{2}\left(\frac{p^{AF}\left(a,f\right)}{p^{A}\left(a\right)p^{F}\left(f\right)}\right),\\ \text{M}{\text{I}}^{BF}=\overset{L}{\underset{f=0}{\sum }}\overset{L}{\underset{b=0}{\sum }}p^{BF}\left(b,f\right)\text{lo}{\text{g}}_{2}\left(\frac{p^{BF}\left(b,f\right)}{p^{B}\left(b\right)p^{F}\left(f\right)}\right), \end{array}$$

where MI^*xF*^ denotes the normalized mutual information between the fused image and the input image *A*, *B*; *a*, *b* and *f* ∈ [0, *L*], and *L* is the number of bins. *p* ^*A*^(*a*), *p* ^*B*^(*b*), and *p* ^*F*^(*f*) are the normalized gray level histograms of source images and fused image. *p* ^*xF*^(*a*, *f*) is the joint gray level histograms between fused image and each source image.

MI can indicate how much information the fused image *F* conveys about the source images *A* and *B* [22]. Therefore, the greater the value of MI, the better the fusion effect.

#### 4.1.4. Cross Entropy

The cross entropy is defined as [8]:

(12) $$\begin{array}{c} \text{CE}=\overset{L-1}{\underset{l=0}{\sum }}P_{l}\;\text{lo}{\text{g}}_{2}\frac{P_{l}}{Q_{l}}, \end{array}$$

where *P*_*l*_ and *Q*_*l*_ denote the gray level histogram of the source image and the fused image, respectively. The cross entropy is used to evaluate the difference between the source images and the fused image. The lower value corresponds to the better fusion result.

#### 4.1.5. Spatial Frequency

Spatial frequency is defined as [40]:

(13) $$\begin{array}{c} \text{SF}=\sqrt{\text{R}{\text{F}}^{2}+\text{C}{\text{F}}^{2}}, \end{array}$$

where RF and CF are the row frequency and column frequency, respectively, and are defined as

(14) $$\begin{array}{c} \text{RF}=\sqrt{\frac{1}{M_{0}N_{0}}\overset{M_{0}-1}{\underset{i=0}{\sum }}\overset{N_{0}-1}{\underset{j=0}{\sum }}{\left[F\left(i,j\right)-F\left(i,j-1\right)\right]}^{2}},\\ \text{CF}=\sqrt{\frac{1}{M_{0}N_{0}}\overset{M_{0}-1}{\underset{i=0}{\sum }}\overset{N_{0}-1}{\underset{j=0}{\sum }}{\left[F\left(i,j\right)-F\left(i-1,j\right)\right]}^{2}}. \end{array}$$

The spatial frequency reflects the edge information of the fused image. Larger spatial frequency values indicate better image quality.

### 4.2. Experiments on Multimodal Medical Image Fusion

**Figure 4:**
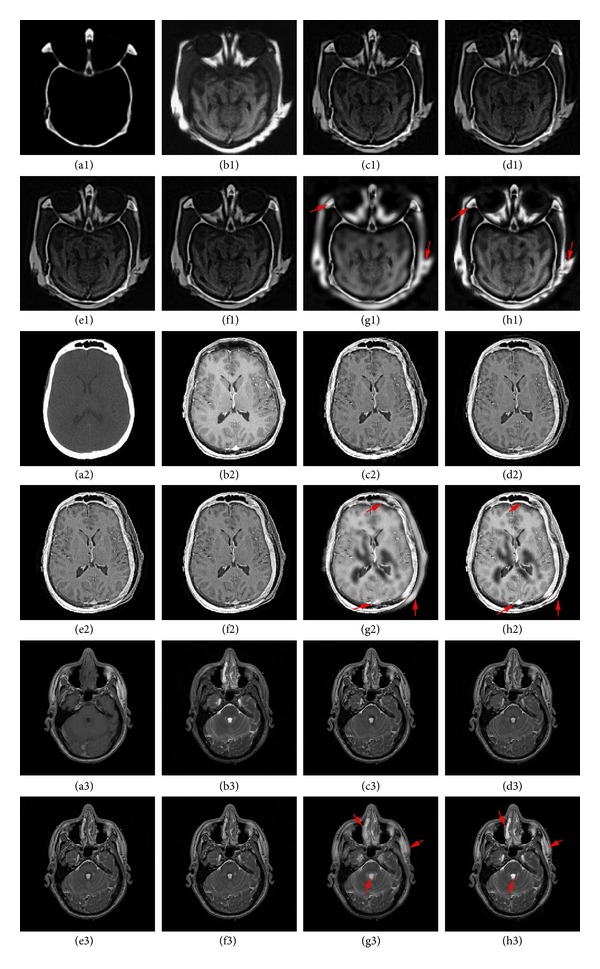
Source multimodal medical images: (a1), (b1) image group 1 (CT and MRI); (a2), (b2) image group 2 (CT and MRI); (a3), (b3) image group 3 (MR-T1 and MR-T2); fused images from (c1), (c2), (c3) DWT based method; (d1), (d2), (d3) FDCT based method; (e1), (e2), (e3) DTCWT based method; (f1), (f2), (f3) NSCT-1 based method; (g1), (g2), (g3) NSCT-2 based method; (h1), (h2), (h3) our proposed method.

To evaluate the performance of the proposed image fusion approach, the experiments are performed on three groups of multimodal medical images. These images are characterized in two distinct pairs: (1) CT and MRI; (2) MR-T1 and MR-T2. The images in Figures 4(a1)-4(b1) and 4(a2)-4(b2) are CT and MRI images, whereas Figures 4(a3)-4(b3) are T1-weighted MR image (MR-T1) and T2-weighted MR image (MR-T2). All images have the same size of 256 × 256 pixel, with 256-level gray scale. For all these image groups, the results of the proposed fusion framework are compared with those of the traditional discrete wavelet transform (DWT) [13, 16], the second generation curvelet transform (fast discrete curvelet transform, FDCT) [41, 42], the dual tree complex wavelet transform (DTCWT) [4], and the nonsubsampled Contourlet transform (NSCT-1 and NSCT-2) based methods. The high-frequency coefficients and low-frequency coefficients of DWT, FDCT, DTCWT, and NSCT-1 based methods are merged by the popular widely used fusion rule of selecting the coefficient with larger absolute values and the averaging rule (average-maximum rule), respectively. NSCT-2 based method is merged by the fusion rules proposed by Bhatnagar, et al. in [26]. In order to perform a fair comparison, the source images are all decomposed into the same levels with 3 for those methods except FDCT method. For DWT method, the images are decomposed using the DBSS (2, 2) wavelet. For implementing NSCT, “9-7” filters and “pkva” filters (how to set the filters can be seen in [43]) are used as the pyramidal and directional filters, respectively.

#### 4.2.1. Subjective Evaluation

The first pair of medical images are two groups of brain CT and MRI images on different aspects, shown in Figures 4(a1), 4(b1) and 4(a2), 4(b2), respectively. It can be easily seen that the CT image shows the dense structure while MRI provides information about soft tissues. The obtained fused images from DWT, FDCT, DTCWT, NSCT-1, and NSCT-2 are shown in Figures 4(c1)–4(g1) and 4(c2)–4(g2), respectively. The results for the proposed fusion method have been shown in Figures 4(h1) and 4(h2). On comparing these results, it can be easily observed that the proposed method outperforms those fusion methods and has good visual representation of fused image.

The second pair of medical images are MR-T1 and MR-T2 images, shown in Figures 4(a3) and 4(b3). The comparison of DWT, FDCT, DTCWT, NSCT-1, NSCT-2, and proposed method, shown in Figures 4(c3)–4(h3), clearly implies that the fusion result of the proposed method has better quality and contrast in comparison to other methods.

Similarly, on observing the noticeable improvement has been emphasized in Figure 4 by the red arrows and the analysis above, one can easily verify the fact that again the proposed method has been found superior in terms of visual representation over DWT, FDCT, DTCWT, NSCT-1, and NSCT-2 fusion methods.

#### 4.2.2. Objective Evaluation

**Figure 5:**
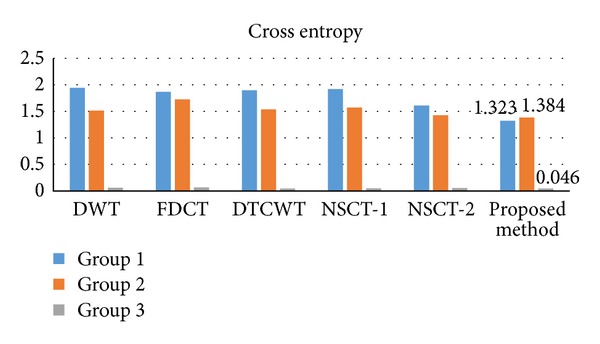
Comparison on cross entropy of different methods and images.

**Figure 6:**
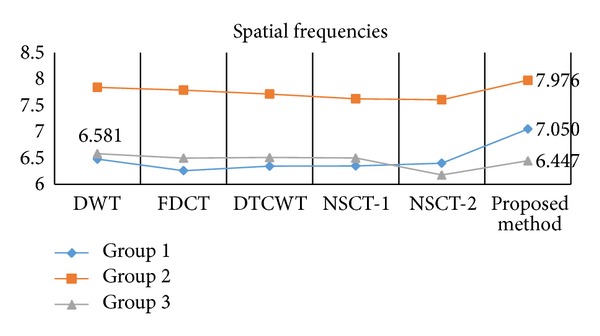
Comparison on spatial frequencies of different methods and images.

For objective evaluation of the fusion results, shown in Figure 4, we have used five fusion metrics: cross entropy, spatial frequency, STD, *Q* ^*AB*/*F*^, and MI. The quantitative comparison of cross entropy and spatial frequency for these fused images is visually given by Figures 5-6 and other metrics are given by Table 1.

On observing Figure 5, one can easily observe all the three results of the proposed scheme have lower values of cross entropy than any of the DWT, FDCT, DTCWT, NSCT-1, and NSCT-2 fusion methods. The cross entropy is used to evaluate the difference between the source images and the fused image. Therefore, the lower value corresponds to the better fusion result.

On observing Figure 6, two values of the spatial frequency of the fused image obtained by the proposed method are the highest, and the other one is 6.447 which is close to the highest value 6.581. Observation of Table 1 yields that all the three results of the proposed fusion scheme have higher values of STD, *Q* ^*AB*/*F*^, and MI than any of other methods except one value of *Q* ^*AB*/*F*^ (image group 2) is the second best. However, an overall comparison shows the superiority of the proposed fusion scheme.

#### 4.2.3. Combined Evaluation

Since the subjective and objective evaluations separately are not able to examine fusion results, we have combined them. From these figures (Figures 4–6) and table (Table 1), it is clearly to find that the proposed method not only preserves most of the source images characteristics and information, but also improves the definition and the spatial quality better than the existing methods, which can be justified by the optimum values of objective criteria except one value of spatial frequency (image group 3) and one value of *Q* ^*AB*/*F*^ (image group 2). Consider the example of the first set of images: the five criteria values of the proposed method are 1.323 (cross entropy), 7.050 (spatial frequency), 58.476 (STD), 0.716 (*Q* ^*AB*/*F*^), and 2.580 (MI), respectively. Each of them is the optimal one in the first set of experiments.

Among these methods, the result of NSCT-2 based method also gives poor results when comparing to the proposed NSCT-based method. This stems from the fact that high-frequency fusion rule of NSCT-2 based method is not able to extract the detail information in the high frequency effectively. Also, by carefully looking at the outputs of the proposed NSCT-based method (Figures 4(h1), 4(h2), and 4(h3)), we can find that they get more contrast and more spatial resolution than the outputs of NSCT-2 based method (highlighted by the red arrows) and other methods. The main reason behind the better performance is that the proposed fusion rules for low- and high-frequency coefficients can effectively extract prominent and detail information from the source images. Therefore, it can be possible to conclude that the proposed method is better than the existing methods.

### 4.3. Fusion of Multimodal Medical Noisy Images and a Clinical Example

**Figure 7:**
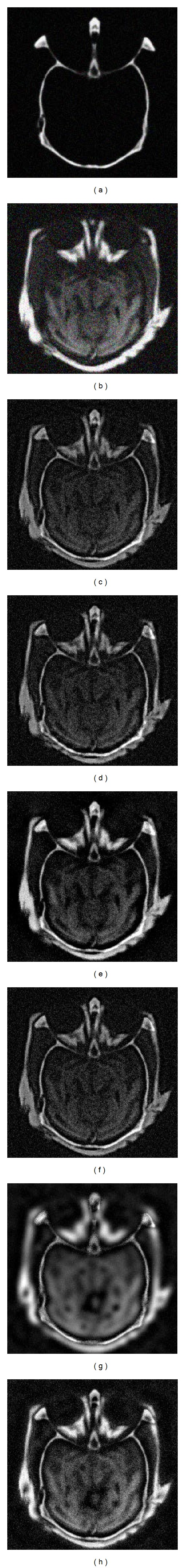
The multimodal medical images with noise: (a) CT image with 5% noise, (b) MRI image with 5% noise; fused images by (c) DWT, (d) FDCT, (e) DTCWT, (f) NSCT-1, (g) NSCT-2, and (h) proposed method.

To evaluate the performance of the proposed method in noisy environment, the input image group 1 has been additionally corrupted with Gaussian noise, with a standard deviation of 5% (shown in Figures 7(a) and 7(b)). In addition, a clinical applicability on noninvasive diagnosis of neoplastic disease is given in the last subsection.

#### 4.3.1. Fusion of Multimodal Medical Noisy Images

For comparison, apart from visual observation, objective criteria on STD, MI, and *Q* ^*AB*/*F*^ are used to evaluate how much clear or detail information of the source images is transferred to the fused images. However, maybe these criteria cannot effectively evaluate the performance of the fusion methods in terms of the noise transmission. For further comparison, Peak Signal to Noise Ratio (PSNR), a ratio between the maximum possible power of a signal and the power of noise that affects the fidelity [44], is used. The larger the value of PSNR, the less the image distortion [45]. PSNR is formulated as

(15) $$\begin{array}{c} \text{PSNR}=10\text{lg}\left|\frac{255^{2}}{\text{RMS}{\text{E}}^{2}}\right|, \end{array}$$

where RMSE denotes the Root Mean Square Error between the fused image and the reference image. The reference image in the following experiment is selecting from Figure 4(h1), which is proven to be the best performance compared to other images.

**Figure 8:**
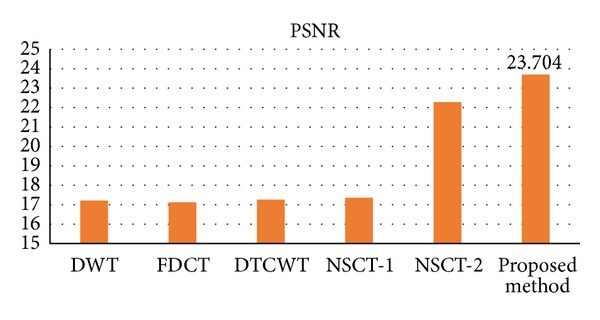
Comparison on PSNR of different methods for the noise medical images.

Figure 7 illustrates the fusion results obtained by the different methods. The comparison of the images fused by DWT, FDCT, DTCWT, NSCT-1, NSCT-2, and proposed method, shown in Figures 7(c)–7(h), clearly implies that the fused image by proposed method has better quality and contrast than other methods.  Figure 8 shows the values of PSNR of different methods in fusing noisy images. One can observe that the proposed method has higher values of PSNR than any of the DWT, FDCT, DTCWT, NSCT-1, and NSCT-2 fusion methods.  Table 2 gives the quantitative results of fused images and shows that the values of STD, *Q* ^*AB*/*F*^, and MI are also the highest of all the six methods. From the analysis above, we can also observe that the proposed scheme provides the best performance and outperforms the other algorithms. In addition, compared with the result of the NSCT-1 method using the average-maximum rule, it demonstrated the validities of the proposed fusion rule in noisy environment.

#### 4.3.2. A Clinical Example on Noninvasive Diagnosis

**Figure 9:**
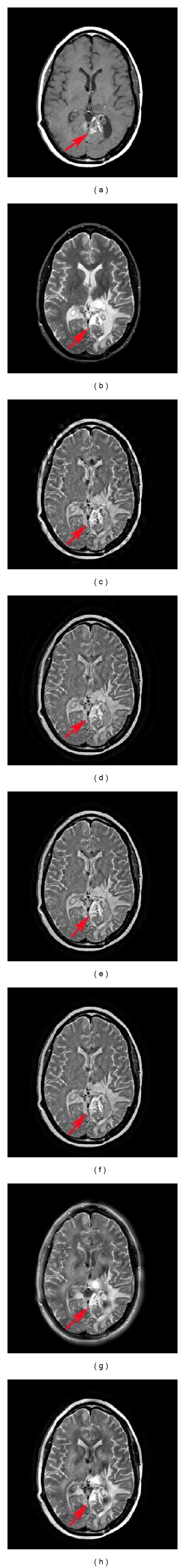
Brain images of the man with recurrent tumor: (a) MR-T1 image, (b) MR-T2 image; fused images by (c) DWT, (d) FDCT, (e) DTCWT, (f) NSCT-1, (g) NSCT-2, and (h) proposed method.

In order to demonstrate the practical value of the proposed scheme in medical imaging, one clinical case on neoplastic diagnosis is considered where MR-T1/MR-T2 medical modalities are used. The images have been downloaded from the Harvard University site (http://www.med.harvard.edu/AANLIB/home.html). Figures 9(a)-9(b) show the recurrent tumor case of a 51-year-old woman who sought medical attention because of gradually increasing right hemiparesis (weakness) and hemianopia (visual loss). At craniotomy, left parietal anaplastic astrocytoma was found. A right frontal lesion was biopsied. A large region of mixed signal on MR-T1 and MR-T2 images gives the signs of the possibility of active tumor (highlighted by the red arrows).

Figures 9(c)–9(h) show the fused images by DWT, FDCT, DTCWT, NSCT-1, NSCT-2, and proposed method. It is obvious that the fused image by proposed method has better contrast and sharpness of active tumor (highlighted by the red arrows) than other methods.  Table 3 shows the quantitative evaluation of different methods for the clinical medical images. The values of the proposed method are optimum in terms of STD, MI, and *Q* ^*AB*/*F*^. From Figure 9 and Table 3, we can obtain the same conclusion that the proposed scheme provides the best performance and outperforms the other algorithms.

## 5. Conclusions

Multimodal medical image fusion plays an important role in clinical applications. But the real challenge is to obtain a visually enhanced image through fusion process. In this paper, a novel and effective image fusion framework based on NSCT and Log-Gabor energy is proposed. The potential advantages include (1) NSCT is more suitable for image fusion because of its advantages such as multiresolution, multidirection, and shift-invariance; (2) a new couple of fusion rules based on phase congruency and Log-Gabor energy are used to preserve more useful information in the fused image to improve the quality of the fused images and overcome the limitations of the traditional fusion rules; and (3) the proposed method can provide a better performance than the current fusion methods whatever the source images are clean or noisy. In the experiments, five groups of multimodal medical images, including one group with noise and one group clinical example of a woman affected with recurrent tumor, are fused by using traditional fusion methods and the proposed framework. The subjective and objective comparisons clearly demonstrate that the proposed algorithm can enhance the details of the fused image and can improve the visual effect with less information distortion than other fusion methods. In the future, we plan to design a pure C++ platform to reduce the time cost and extend our method for 3D or 4D medical image fusion.

## Figures and Tables

**Table 1:** Comparison on quantitative evaluation of different methods for the first set medical images.

Images	Evaluation	DWT	FDCT	DTCWT	NSCT-1	NSCT-2	Proposed method
Image group 1 (CT and MRI)	STD	41.160	39.170	40.132	40.581	54.641	**58.476**
	Q^AB/F^	0.618	0.592	0.652	0.683	0.427	**0.716**
	MI	2.371	2.029	2.393	2.438	1.886	**2.580**

Image group 2 (CT and MRI)	STD	72.260	70.572	71.407	71.733	80.378	**83.334**
	Q^AB/F^	0.567	0.584	0.606	**0.622**	0.450	0.618
	MI	3.137	3.138	3.198	3.235	2.915	**3.302**

Image group 3 (MR-T1 and MR-T2)	STD	37.358	36.856	37.358	37.277	39.023	**40.630**
	Q^AB/F^	0.635	0.647	0.668	0.682	0.589	**0.689**
	MI	3.209	3.142	3.302	3.347	3.349	**3.469**

**Table 2:** Comparison on quantitative evaluation of different methods for the noise medical images.

Evaluation	DWT	FDCT	DTCWT	NSCT-1	NSCT-2	Proposed method
STD	41.893	40.044	40.995	41.338	52.533	**57.787**
Q^AB/F^	0.592	0.579	0.619	0.637	0.269	**0.640**
MI	2.785	2.539	2.826	2.882	1.816	**2.914**

**Table 3:** Comparison on quantitative evaluation of different methods for the clinical medical images.

Evaluation	DWT	FDCT	DTCWT	NSCT-1	NSCT-2	Proposed method
STD	64.433	62.487	63.194	63.652	60.722	**67.155**
Q^AB/F^	0.541	0.565	0.578	0.597	0.411	**0.624**
MI	2.507	2.455	2.540	2.584	2.392	**2.624**
